# Tirofiban-Induced Diffuse Alveolar Hemorrhage in a Patient With Acute Myocardial Infarction Following Primary Percutaneous Coronary Intervention: A Case Report

**DOI:** 10.7759/cureus.95855

**Published:** 2025-10-31

**Authors:** Xiaoyan Li, Guoqing Qi, Liye Wei

**Affiliations:** 1 Department of Cardiology, The First Hospital of Hebei Medical University, Shijiazhuang, CHN

**Keywords:** acute myocardial infarction, complication of treatment, diffuse alveolar hemorrhage, percutaneous coronary intervention infarction, tirofiban

## Abstract

This case report presents a rare instance of tirofiban-induced diffuse alveolar hemorrhage (DAH) in a 66-year-old male undergoing primary percutaneous coronary intervention for acute myocardial infarction. Approximately 22 hours after tirofiban initiation, the patient developed acute dyspnea and hemoptysis. Imaging studies revealed bilateral alveolar infiltrates consistent with DAH, accompanied by a significant drop in hemoglobin. Cessation of tirofiban and dual antiplatelet therapy led to rapid clinical improvement within 24 hours, with complete radiological resolution observed by day 15. Other potential etiologies were ruled out through serological testing. This case highlights DAH as a serious, albeit rare, complication of glycoprotein IIb/IIIa inhibitor therapy and emphasizes the importance of early recognition and immediate drug discontinuation to ensure favorable outcomes.

## Introduction

Acute myocardial infarction (AMI) remains a leading cause of morbidity and mortality worldwide, with timely revascularization through primary percutaneous coronary intervention (PCI) being the cornerstone of management. To minimize the risk of acute stent thrombosis and recurrent ischemic events, adjunctive antiplatelet therapy is essential. Among these agents, glycoprotein IIb/IIIa inhibitors (GPIs), such as tirofiban, are frequently administered in high-risk PCI scenarios due to their potent inhibition of platelet aggregation. By binding to the GP IIb/IIIa receptor on platelets, tirofiban prevents fibrinogen cross-linking and thus thrombus formation, significantly reducing peri-procedural ischemic complications [1].

However, the potent antiplatelet effect of GPIs is also associated with an increased risk of bleeding. Major bleeding events, although relatively uncommon, can have serious clinical implications, including increased mortality. While access-site bleeding and gastrointestinal hemorrhages are more frequently reported, pulmonary complications such as diffuse alveolar hemorrhage (DAH) are exceedingly rare and less commonly recognized [2]. DAH is a life-threatening condition characterized by widespread bleeding into the alveolar spaces, leading to impaired gas exchange and potential respiratory failure. Although most commonly associated with autoimmune diseases, infections, or other vasculitic syndromes, drug-induced DAH, particularly related to antiplatelet agents, has been increasingly documented in recent years [3].

This case report contributes to the limited body of literature on tirofiban-associated DAH, underscoring the necessity for heightened clinical vigilance when using potent antiplatelet agents, especially in patients undergoing complex coronary interventions.

## Case presentation

A 66-year-old male presented to the emergency department 60 minutes after the onset of chest pain radiating to the left arm. Admission electrocardiography revealed ST-segment elevation in leads II, III, aVF, and V7-V9, consistent with acute inferoposterior myocardial infarction. After receiving 300 mg of aspirin and 600 mg of clopidogrel, the patient underwent primary PCI. Coronary angiography demonstrated subtotal thrombotic occlusion of the proximal right coronary artery, which was treated with thrombus aspiration and drug-eluting stent implantation. The patient received a tirofiban bolus (10 μg/kg over three minutes) followed by a continuous infusion (0.15 μg/kg/min).

**Figure 1:**
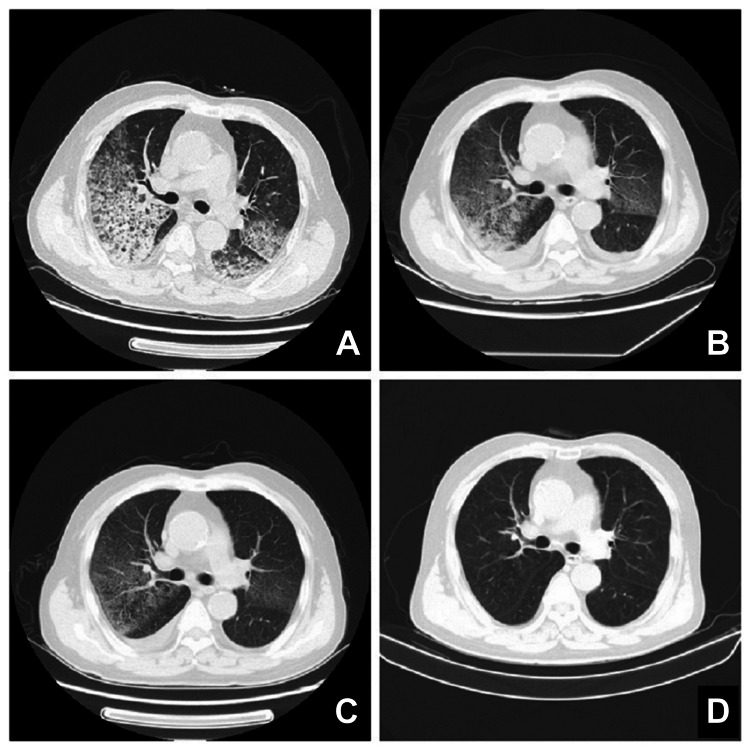
Serial chest computed tomography (CT) scans of a 66-year-old man with acute myocardial infarction. (A) Widespread new bilateral alveolar infiltrates 22 hours after tirofiban administration (diagnosis of DAH). (B) Reduced bilateral infiltrates on day five after tirofiban discontinuation. (C) Further resolution of infiltrates on day 10. (D) Complete clearance of infiltrates on day 15.

Approximately 22 hours after initiating tirofiban, he developed dyspnea and hemoptysis with bright red blood. Chest computed tomography (CT) revealed diffuse bilateral alveolar infiltrates, confirming DAH (Figure 1A). Hemoglobin decreased from 138 g/L to 93 g/L. Platelet count and activated partial thromboplastin time (APTT) remained normal. Arterial blood gas analysis showed PaO₂ 62.9 mmHg, PaCO₂ 25 mmHg, and pH 7.45. Tirofiban and dual antiplatelet therapy (DAPT) were discontinued immediately. Oxygen therapy was administered via facemask. Serial platelet counts and coagulation profiles remained normal. Serological tests for ANCA (antineutrophil cytoplasmic antibodies), anti-GBM antibodies, antinuclear antibody (ANA), and anti-phospholipid antibodies were negative. Clinical symptoms improved within 24 hours of stopping tirofiban. DAPT (aspirin and clopidogrel) was cautiously restarted on day three to prevent acute stent thrombosis. Oxygenation progressively improved, and follow-up chest CT demonstrated resolution of bilateral infiltrates over subsequent days (Figures 1B, 1C). Complete clinical and radiological resolution was achieved by day 15 (Figure 1D). The patient was discharged without further complications.

## Discussion

Tirofiban, a selective non-peptide GP IIb/IIIa inhibitor, is widely used in contemporary cardiology practice for its rapid onset and short half-life, allowing for rapid reversal of its effects upon discontinuation. Despite its benefits in reducing ischemic events, tirofiban carries a recognized risk of hemorrhage, with DAH representing one of its most severe, though infrequent, complications [4]. The exact mechanism underlying tirofiban-induced DAH remains incompletely elucidated. It is hypothesized that profound platelet inhibition may disrupt alveolar-capillary integrity, facilitating erythrocyte extravasation into the alveolar space. Additionally, individual susceptibility, possibly influenced by genetic polymorphisms in platelet receptors or underlying subclinical pulmonary pathology, may contribute [5].

In the present case, the temporal relationship between tirofiban initiation and symptom onset, along with the exclusion of other common causes of DAH through comprehensive serological testing, strongly supports a drug-induced etiology. The rapid clinical improvement following drug withdrawal further corroborates this association. Notably, the patient did not require corticosteroid therapy, which is often employed in immune-mediated DAH but may not be necessary in drug-induced cases where removal of the offending agent suffices [6].

This case also highlights the importance of multidisciplinary management involving cardiology, pulmonology, and critical care teams. Early recognition of respiratory symptoms such as dyspnea and hemoptysis in a patient receiving GPIs should prompt immediate evaluation, including imaging and hematologic assessment. Cessation of all antithrombotic agents, however transient, must be balanced against the risk of stent thrombosis. In this instance, DAPT was cautiously reintroduced after clinical stabilization, without recurrence of hemorrhage.

Previous case reports and small series have documented similar episodes with other GPIs, including abciximab and eptifibatide, suggesting a class effect [7,8]. Thus, clinicians should be aware of this potential complication across all GPIIb/IIIa inhibitors. Furthermore, ongoing education and awareness are crucial for prompt diagnosis and intervention, which are key to favorable outcomes.

In conclusion, while tirofiban is an effective agent in preventing thrombotic complications during PCI, clinicians must remain vigilant for rare but serious adverse effects such as DAH. Early detection, immediate drug discontinuation, and supportive care are essential steps in managing this condition and ensuring patient safety.

## Conclusions

Tirofiban, though effective in preventing thrombotic events during PCI, may rarely cause DAH, a severe complication requiring high clinical vigilance. Immediate recognition of respiratory distress and hemoptysis, coupled with prompt drug discontinuation, is essential. This case demonstrates that early intervention can lead to rapid recovery without sequelae. Physicians should be aware of this potential adverse effect to ensure timely diagnosis and management.
